# Critical features of peer assessment of clinical performance to enhance adherence to a low back pain guideline for physical therapists: a mixed methods design

**DOI:** 10.1186/s12909-015-0484-1

**Published:** 2015-11-12

**Authors:** Marjo J. M. Maas, Simone A. van Dulmen, Margaretha H. Sagasser, Yvonne F. Heerkens, Cees P. M. van der Vleuten, Maria W. G. Nijhuis-van der Sanden, Philip J. van der Wees

**Affiliations:** 1 HAN University of Applied Sciences, Institute for Health Studies, Kapittelweg 33, 5425 EN Nijmegen, The Netherlands; 2 Radboud University Medical Center, Institute for Health Sciences, IQ healthcare, Geert Grooteplein 21, 6525 EZ Nijmegen, The Netherlands; 3 Radboud University Medical Center Department of Primary and Community Care, Nijmegen, The Netherlands; 4 Dutch Institute of Allied Health Care, Amersfoort, The Netherlands; 5 Maastricht University, Department of Educational Development and Research, Faculty of Health, Medicine and Life Sciences, Maastricht, The Netherlands

**Keywords:** Peer assessment, Peer review, Guideline adherence, Knowledge translation, Performance assessment

## Abstract

**Background:**

Clinical practice guidelines are intended to improve the process and outcomes of patient care. However, their implementation remains a challenge. We designed an implementation strategy, based on peer assessment (PA) focusing on barriers to change in physical therapy care. A previously published randomized controlled trial showed that PA was more effective than the usual strategy “case discussion” in improving adherence to a low back pain guideline. Peer assessment aims to enhance knowledge, communication, and hands-on clinical skills consistent with guideline recommendations. Participants observed and evaluated clinical performance on the spot in a role-play simulating clinical practice. Participants performed three roles: physical therapist, assessor, and patient. This study explored the critical features of the PA program that contributed to improved guideline adherence in the perception of participants.

**Methods:**

Dutch physical therapists working in primary care (*n* = 49) organized in communities of practice (*n* = 6) participated in the PA program. By unpacking the program we identified three main tasks and eleven subtasks. After the program was finished, a questionnaire was administered in which participants were asked to rank the program tasks from high to low learning value and to describe their impact on performance improvement. Overall ranking results were calculated. Additional semi-structured interviews were conducted to elaborate on the questionnaires results and were transcribed verbatim. Questionnaires comments and interview transcripts were analyzed using template analysis.

**Results:**

Program tasks related to performance in the therapist role were perceived to have the highest impact on learning, although task perceptions varied from challenging to threatening. Perceptions were affected by the role-play format and the time schedule. Learning outcomes were awareness of performance, improved attitudes towards the guideline, and increased self-efficacy beliefs in managing patients with low back pain. Learning was facilitated by psychological safety and the quality of feedback.

**Conclusion:**

The effectiveness of PA can be attributed to the structured and performance-based design of the program. Participants showed a strong cognitive and emotional commitment to performing the physical therapist role. That might have contributed to an increased awareness of strength and weakness in clinical performance and a motivation to change routine practice.

**Electronic supplementary material:**

The online version of this article (doi:10.1186/s12909-015-0484-1) contains supplementary material, which is available to authorized users.

## Background

Clinical practice guidelines are intended to optimize patient care and improve patient outcomes [1]. Guidelines are also increasingly regarded as a part of professional quality systems and policies [2]. However, the uptake of guidelines in physical therapy (PT) practice remains a challenge, despite the variety of implementation strategies that have been developed [3–5]. Professionals are hampered by a lack of commitment to the guidelines, insufficient knowledge and skills related to the guidelines, and limited social and organizational support [6–8]. In addition, a study by Rutten et al. [9] on determinants of guideline adherence showed that physical therapists (PTs) do not hold realistic perceptions of the extent to which they adhere to guideline recommendations.

The limited ability of clinicians to accurately self-assess the quality of their professional performance is not new [10]. A compelling body of research evidence shows that the development of adequate self-perception requires both internal and external information about one’s professional performance, including appropriate performance standards [11–15]. There is a need for interventions containing feedback that can help to develop realistic self-perceptions of guideline adherent behavior and enhance motivation to change routine practice.

We designed an implementation strategy based on peer assessment (PA) that targets identified barriers to change for PTs in primary care [16]. We tailored an existing PA design that was shown to be effective in undergraduate PT education [17] to the context of professional PT practice and to the purpose of guideline implementation. In a previously published randomized-controlled trial (Table 1), PA was shown to be more effective than the traditional “case discussion” implementation strategy [18]. We analyzed this PA program to determine the critical features of its success.

**Table 1:** Overview of the methods and results of a previously published trial (Van Dulmen et al. [18])

Design
A cluster-randomized controlled trial was conducted among 10 communities of practice (CoPs) of Dutch physical therapists (*n* = 90) to compare the effectiveness of two implementation strategies: peer assessment (PA) and case discussion (CD). Both strategies aimed to improve adherence to the clinical practice guidelines for the management of patients with low back pain. The programs consisted of four meetings over a six-month period. Outcomes were measured at baseline and at 6 months follow up.
Randomization and intervention allocation
CoPs showing interest in the program were invited to a plenary meeting in November 2009. They were informed that the study compared two educational strategies, and that both programs required an equal amount of time and effort. All physical therapists regularly treating patients with low back pain were eligible for inclusion. Included CoPs were randomly allocated to the PA group and the CD group resulting in six CoPs for the PA program (*n* = 49) and four CoPs for the CD program (*n* = 41).
Interventions
PA is the process whereby professionals evaluate or are being evaluated by their peers and provide each other with performance feedback. The main difference between PA and CD is that in the PA approach the tasks were structured, with a focus on performance rather than discussion, and participant roles were pre-defined. In the CD approach the tasks were less structured with ample opportunity for in-depth elaboration and discussion, and participant roles were not defined. In PA and CD, participants worked on identical cases concerning problem content, but for PA these cases were adjusted to allow for performance of participants in different roles. In PA, written cases were not known in advance but were presented by a coach on the spot, simulating daily clinical practice. For CD groups, written cases were included in the program guide to allow for proper preparation, along with instructions and written questions to guide the discussion process.
Outcome measures
Outcomes were assessed at baseline and at six months. Primary outcome was knowledge and guideline-consistent reasoning, measured with 12 performance indicators using four vignettes that fully covered the patient profiles described in the guidelines. Changes in reflective practice were measured with the Self-Reflection and Insight Scale (Grant et al., [49]).
Results
Vignettes were completed by 78 participants (PA group *n* = 44; CD group *n* = 34). Multilevel analysis showed an increase in guideline-consistent clinical reasoning of 8.4 % in the peer-assessment groups whereas the control groups showed a decline of 0.1 % (estimated group difference 8.7 %; 95 % CI: 3.9 to 13.4; *P* < 0.001). No group differences were found for self-reflection.

In PA professionals evaluate or are being evaluated by observing their peers in a role-play that simulates PT practice. They provide each other with performance feedback that might evoke reflection and identify areas of clinical performance that need improvement [19, 20]. Personal assumptions about one’s professional competence can be compared with peer views that might compensate for poor self-assessment [13, 14]. Peer assessment enhances the development of a mutually accepted quality standard of performance by introducing peers to an “assessor” or “auditor” perspective [23, 26]. In this respect, PA might be a an effective tool to enhance bottom up quality improvement and accountability of health care [21, 22].

Research shows that effective peer assessment practices are context-specific and culture dependent [23, 24], and these findings also apply to effective implementation strategies [25]. Thus, to enhance the generalizability of the trial results, and to allow for adequate knowledge transfer, understanding of the causal mechanisms of PA is necessary [25–27].

The aim of this study was to explore the features of the PA program that were perceived to have a powerful impact on learning and change of routine practice.

Our research question was:

*Which elements of the PA program were perceived to have a strong impact on clinical performance improvement consistent with clinical guidelines, and why?*

## Methods

### Study design

We conducted a mixed-methods study using questionnaires and semi-structured interviews to explore the critical features of the PA program that contributed to improved guideline adherence.

### Setting and participants

The Royal Dutch Society for Physical Therapy offers annual professional development programs for the approximately 800 communities of practice in the Netherlands. Communities of practice are small groups of 5-15 PTs who share the same setting or the same interests. The current study focused on communities of practice (*n* = 6; 49 participants) that participated in a randomized controlled trial (Table 1) and were allocated to the PA-condition.

### The peer assessment program

The PA program was launched in February 2010 and finished in September 2010. Its design was built on a mix of theoretical constructs related to learning and professional behavior change, which were assumed to contribute to improved clinical performance [26]. Table 2 shows the theoretical framework, the underlying constructs, and the operationalization of these constructs in the PA design.

**Table 2:** Theoretical framework of the PA program design

Theory	Underlying constructs used	Operationalization of constructs
Social constructivist learning theory [48]	Contextual learning, collaborative learning, active participation, and knowledge construction to enhance attention, storage, and retrieval of knowledge from memory.	Presenting a variety of clinical problems that adequately reflect authentic clinical practice, accounting for the case-specifity of clinical competence.
		Simulating the context of daily practice in a role-play accounting for the context-specifity of clinical competence.
		Enhancing active participation of each participant by assigning pre-defined roles, and by using a performance based format.
Self-regulated learning theory [50, 51]	Applying metacognitive strategies to guide the professional development process.	
	Self-assessment	Designing an improvement plan based on peer feedback.
	Conscious goal setting and action planning	Discussing the improvement plan with peers.
Situated learning theory [40, 52]	Learning in the context of daily practice to bridge the gap between learning context and application context.	Delivering the program within communities of practice that share the same setting or the same interest.
Social cognitive learning theory [33]	Enhancing the development of self-efficacy beliefs.	
	Performing the new behavior and experiencing the consequences of that behavior (mastery experience).	Performing the new behavior individually, by reasoning aloud and demonstrating diagnostic and treatment skills relevant to the LBP guidelines.
	Observing the behavior of others and the consequences of that behavior (vicarious experience).	Observing a peer’s performance and providing individualized improvement feedback.
Stages of change theory [53]	Alligning implementation strategies to the stages of change.	Delivering the program within communities of practice. Peers are involved in the professional development process and are capable of tailoring feedback to stages of change.
Theory of planned behaviour [34]	Changing attitudes and subjective norms toward the new behavior.	Introducing peers to the assessor perspective. In appraising a peers’ performance, peer assessors need to develop an understanding and a mutually accepted quality standard to deliver credible performance feedback.
	Enhancing the development of self-effecacy beliefs.	

The PA program aimed to enhance clinical performance consistent with guideline recommendations including knowledge, communication, and hands-on clinical skills. Clinical performance was directly observed and evaluated by peers in a role-play that simulated clinical practice. Participants received a PA-manual in advance, containing a description of the PAprocedure, a time schedule for each meeting, and guidelines for receiving and providing constructive feedback. They received a link to the updated guideline “Low back pain for physical therapy and manual therapy” (Staal et al. [28]) published by the Royal Dutch Society for Physical Therapy. Four meetings were scheduled over a period of six months. As the PTs were novices in the PA method, and no additional training was provided, the PA process was supported by a coach (MM or HE). Coaches were experienced PTs, teachers in PT education, and trained in the PA procedure. They facilitated the process of providing and receiving feedback, and they gave additional feedback when needed.

Each participant performed three roles: PT, assessor and simulated patient. In the PT role, participants completed a written assignment that contained a clinical case and brief instructions for diagnosis or treatment. Clinical cases were developed by a team of experienced PTs and guideline experts. The cases fully covered the patient profiles of LBP described in the guidelines, including red flags. PTs analyzed the clinical cases by reasoning aloud and demonstrated (hands-on) skills relevant to the clinical problem. Afterwards, they reflected on their performance. In the assessor role, peer performance was observed and assessed with a scoring sheet containing performance criteria that could be scored on a 7-point scale (1 = much improvement needed, to 7 = no improvement needed) and space for written feedback. Performance categories addressed diagnosis, treatment, and evaluation. In the patient role, participants received the clinical case along with written simulation instructions. Simulation instructions consisted of a description of the patient’s complaints, including personal factors (e.g., cognitive / emotional), and contextual factors (e.g. family, work) that might be relevant to the patient’s problem. Participants were instructed to improvise patient responses and provide feedback from the patient perspective.

Prior to the third session, each participant developed a personal change plan, including an action plan, based on performance feedback and self-assessment. In the third meeting, the group reviewed change plans and provided additional peer feedback. The fourth session was identical to the first two sessions, but the design of the clinical cases was tailored to participants’ specific learning needs.

### Questionnaires and interviews

Prior to data collection, we unpacked the PA program and identified three main tasks and eleven subtasks that were assumed to affect guideline adherence. Task analysis was supported by guidelines described by Janssen-Noordman et al. [29]. An online questionnaire was administered after completion of the PA program in which participants were asked to rank the program tasks from high to low learning value, assigning the highest rank for the most learning value and the lowest rank for the least. Subsequently, they were asked to provide written comments on the three most instructive PA task elements (Additional file 1).

Emerging questions from the questionnaires comments served as input for conducting semi-structured interviews to obtain more understanding of how the PA program affected professional development (Additional file 2). In contrast to a reductionist approach to the data by means of task analysis and task ranking, the interviews had a more holistic approach, focusing on experiences with the PA program as an integrated system. From each peer group, one PT was selected for an interview (*n* = 6). Purposeful selection was based on average and deviant ranking results. An interview guide was designed by MM and PW addressing the three main questions that emerged from the questionnaire data:

1. What did you expect of the PA program?
2. How did you perceive the PA program, and how did it affect your daily practice?
3. In the questionnaire, you indicated that you perceived task X, Y and Z to have the strongest learning value. Can you explain why?

Selected participants were invited by e-mail, and received information about the study’s purpose, procedure, the use of the data, and the focus of the interview.

The first interview was conducted by MM and PW face-to-face. The following interviews were conducted by either MM or PW using teleconferencing technology. To enhance the credibility of the results, research assistants AS and GB joined the telephone interviews, taking notes and posing additional questions when needed. Interviews of each participant, including verbal consent, were audiotaped. Interviews lasted between 45 and 90 minutes. Recordings were transcribed verbatim. An independent check on the transcripts was conducted by AS and GB.

## Data analysis

### Quantitative analysis

Ranking results were described by calculating mean, median, and sum scores for each learning task using IBM SPSS statistics 20.

### Qualitative analyses

A sample of texts from questionnaires and interview transcripts was studied and coded by MM and PW independently. The analytic process was guided by template analysis that combines a-priori codes with emerging codes [30]. The PA program as a whole and its learning tasks and subtasks served as a-priori codes. Additional codes were defined during the analytic process when these seemed relevant regarding the research question. Codes were compared, and some codes were merged into higher-order codes. PW and MM discussed a codebook until consensus was reached. Subsequently, all written comments in the questionnaires and interview transcripts were analyzed line-by-line, using ATLAS-ti v.7 software. Emerging themes were identified by constant comparison of codes and higher order codes. We summarized the results in a matrix that crossed a-priori codes (tasks and subtasks) and emerging themes from the data [31]. Two independent researchers SD (health scientist and PT) and MS (educational scientist) evaluated the analysis process and outcomes. They were not involved in the design or delivery of the PA program. Disagreements were discussed until consensus was reached and we finally agreed that the matrix fully fitted the data.

### Ethical aspects

This project received approval of the medical ethical committee of Radboud University Medical Center. All participants volunteered to participate and gave their informed consent. We adhered to the RATS guidelines for qualitative research [32].

## Results

In total, 44 PTs have finished the program. Table 3 shows an overview of the participants’ characteristics. Two PTs did not fully complete the ranking procedure and were excluded from quantitative analyses (response rate = 86 %). All PTs invited for additional interviews (*n* = 6) agreed to participate.

**Table 3:** Peer assessment group characteristics

Physical therapist characteristics	*N* = 44
Age mean (SD)	40.4 (12.4)
Sex (male/female)	17/27
Working hours per week (SD)	32.5 (9.6)
Treatment of patients with LBP per year	
<25	12
25-50	12
50-75	6
76-100	5
>100	10
Manual therapist	8
Years of experience (SD)	16.5 (11.9)

### Results quantitative analysis

Ranking results showed that participants committed the most to subtasks related to task performance in the PT role. Receiving peer feedback was perceived as the most valuable element, followed by receiving external coach feedback, performing the clinical task individually*,* and receiving simulated patient feedback. Participants varied widely in their preferences for learning in the PT role, but agreed on the superior value of receiving peer feedback. Table 4 shows an overview of the results.

**Table 4:** Results quantitative analysis

Tasks	Subtasks	Mean	Median	Range	Sum
Study manual	Study PA procedure and guidelines	5.09	6.0	10	195
Perform in PT role	Perform clinical task individually	8.05	9.0	10	322
	Receive peer feedback	9.75	10.0	6	389
	Receive external coach feedback	8.48	9.0	10	331
	Receive simulated patient feedback	6.84	7.0	9	253
	Receive written feedback and scores	2.91	2.0	9	102
Perform in assessor role	Observe peer performance	6.46	6.0	9	252
	Provide oral feedback	5.75	5.5	9	230
	Provide written feedback and scores	2.58	2.0	4	44
Design change plan	Design and discuss change plan	6.38	6.00	10	249
Perform in patient role	Simulate patient problem	3.26	3.0	7	98

### Results qualitative analysis

Five themes emerged from the analysis of the questionnaires comments and the additional interview transcripts. These themes were related to the PA program either as a whole, or related to its specific learning tasks and subtasks: a) general perceptions of the PA program, b) determinants of PA affecting perceptions, c) facilitators for learning, d) learning activities, and e) learning outcomes.

We summarized the results by creating a matrix that crossed a-priori categories (program tasks and subtasks) with emerging themes, leaving empty fields where data were not available (Table 5). Program tasks and subtasks in the matrix follow the build-up of the PA program. In the next section, we first discuss the general perceptions of the PA program, determinants of PA affecting these perceptions, and the general outcomes. Second, we discuss the subtasks by following the matrix, including their related learning activities, outcomes, and facilitators for learning. Although we did not explicitly ask participants to comment on tasks that were perceived as less instructive, they often did so spontaneously:

**Table 5:** Summary of results qualitative analysis

	PA Program tasks and subtasks	Perceptions of the PA program	Determinants of PA affecting perceptions	Facilitators for learning and change	Learning Processes	Learning Outcomes
	PA Program					Change in attitudes toward guidelines.
						Awareness of professional limitations.
1	Study manual					Update of knowledge.
2	Perform task in PT role	Fear to expose professional competence.	Tight time schedule.	Training in the PT role.	Uncovers weakness.	Awareness of gaps in professional performance.
					Reinforces strength.	
					Stimulates reasoning aloud, self-assessment and critical reflection.	
		Challenge of obtaining performance feedback.				Improved self-confidence in arguing for choices.
			Role play format.	Group safety		
3	Receive peer feedback			Peer feedback is concrete, concise, critical and personal.	Reveals strength and weakness in clinical performance.	Improved self-efficacy beliefs in managing LBP^a^ patients.
					Shows improvement areas. Reveals new reasoning perspectives and performance alternatives.	
				Varied group composition		
					Stimulates self-assessment and critical reflection.	
4	Receive simulated patient feedback				Reveals how interventions are perceived from the patient perspective.	
5	Receive external coach feedback			External coach poses challenging questions, guides the PA process, facilitates giving and receiving feedback, provides non-judgmental, concise feedback, monitors the time schedule, maintains group safety.	Reveals new reasoning perspectives and performance alternatives.	
					Stimulates self-assessment and critical reflection.	
6	Receive written feedback and scores				Stimulates self-assessment and critical reflection.	
7	Observe peer performance			Modeling peer performance.	Reveals new reasoning perspectives and performance alternatives.	Improved self-confidence in managing LBP patients.
8	Provide oral feedback			Training in the assessor role.	Triggers being concrete and concise in reasoning aloud.	Shared quality standards of performance.
					Elicits discussion over criteria.	
9	Provide written feedback and scores					
10	Design change plan				Guides improvement process.	
11	Perform task in Simulated patient role					

^a^*LBP* low back pain

*“Receiving feedback from your colleagues provides new insights. You learn from the mistakes you make, or how you can handle them better. I assigned the lowest ranks to ‘receiving and providing scores’ because I think that scores add nothing to the learning process. Moreover not all aspects of performance can be expressed in scores and scores are not objective” (Q-P8).*

We limit the discussion to comments on the most instructive subtasks. Participants’ quotes are coded by information source (Questionnaire = Q; Interview Transcript = IT) and by participant number (P1 – P42) (Table 5).

## The PA program as a whole

### General perceptions

Participants were generally satisfied with the program. They reported that the mix of written cases adequately reflected the problems encountered in daily practice, however, the PA format was new, and was perceived with mixed feelings. Physical therapists were not used to exposing their professional performance for group review. Some participants appraised the PA program as challenging, providing an excellent opportunity to receive performance feedback; others were reluctant to expose their professional competence, triggered by feelings of performance anxiety.

Specific task features (time schedule and role-play format) affected perceived learning opportunities and threats. Participants, who appreciated the task structure, reported that PA allowed them to solve a considerable number of clinical cases in a relatively short time and trained them to be concrete and concise in reasoning aloud in the PT role as well as in the assessor role.

*“The strongest feature of PA was the structure of the meetings. The system of PA was interesting…for example, I appreciated that repeating feedback that was provided by someone else, was not allowed. It’s useless to repeat advice.”(IT-P41)*

Participants who criticized the task structure perceived the timetable as stressful, and as a barrier to in-depth case discussion.

*“Yes, time pressure was a weakness of PA….sometimes the performance evaluation raised questions which could not be addressed in-depth, because you had to skip to a new problem. I would prefer to perhaps discuss fewer cases more extensively”.(IT-P18)*

From the perspective of the assessor, the role-play was appreciated because it allowed implicit behaviors to become explicit. From the perspective of the assessed, the role-play was critically appraised. Some participants believed that it poorly reflected their authentic professional behaviors, and that they underperformed in the PA context.

*“It was hard to perform a clinical examination or treatment in this setting; partly, because the patient is a colleague. It is not like in your own working room. In addition, you consciously think about the decisions you make, because your steps will be evaluated.” (Q-P8)*

### General learning outcomes

The PA program resulted in distinct levels of self-reported behavioral change. Although participants studied the updated guidelines prior to the program and were tested on their knowledge with clinical vignettes, they reported that applying knowledge in the context of PA increased their understanding of the guidelines, and facilitated their use in clinical practice.

“*Yes, you want to work according to the guidelines. Therefore, you need to master them…I realized that I in fact did not fully understand the guidelines for low back pain. I knew vaguely what the content was, but not exactly. I think I have obtained a better understanding of the classification system of patient profiles, and therefore I apply them more frequently in my work*.” (IT-P18)

Participants noticed that working with the guidelines in the context of the PA program changed their attitudes towards the guidelines. In their view, guidelines are often considered as too theoretical and of limited applicability in daily practice.

*“I also noticed that some colleagues perceived the guidelines as less annoying or boring.” (IT-P18)*

Although participants did not explicitly report changes in their management of patient problems, they did report changes in their professional identity and awareness of the limitations of their profession.

*“What clearly emerged from the cases we discussed [in the PA program] was that as a PT we like to help people and it remains questionable if that is always justified? We somehow suffer from an irrepressible desire to help….we’re inclined to always give care, whereas in some cases restraint would be better.” (IT-P14)*

## Performing the PT role

### Performing the clinical task individually

Although some participants initially felt reluctant to move out of their “comfort zone”, they considered exposure of their routine practice as a necessity for quality improvement. They pointed out that the four PA sessions allowed them to cope with anxiety triggers by training in the PT role.

*“Yes, but you need to push yourself sometimes. I mean…I think it's threatening, it's not pleasant at all……but I also know that it is important to bare your buttocks, and look where you go wrong. No pain no gain, that's a bit of the rationale.”(IT-P15)*

Performance in the PT role necessitated reasoning aloud, triggered underpinning clinical decisions, and stimulated the transfer of research evidence to the context of a particular clinical problem. Participants explained that arguing aloud resulted in improved self-confidence in decision-making. They became more aware of their strengths and weaknesses, either by “reflection in action” or by ‘ “reflection on action”.

Exposing professional performance in the PT role was facilitated by perceived group safety.

“*Your colleagues are the people who know you well and who know what your strengths and your weaknesses are. So they may well shoot at you.” (IT-P18)*

### Receiving peer feedback

Although PTs organized in communities of practice discuss clinical cases on a regular basis, they do not have a culture of asking for and providing performance feedback. The opportunity to receive peer feedback was therefore embraced. Participants felt strengthened in areas of clinical performance they mastered, and felt challenged to appraise areas that needed improvement.

*“Receiving peer feedback clearly revealed my strengths and weaknesses. I immediately understood what I needed to work on. And because my strengths were noticed, it was easier to face my weaknesses.” (Q-P7)*

Learning from peer feedback was facilitated by its quality. Participants preferred personalized feedback, that showed involvement with their development process and their personal learning needs, but feedback should also focused.

*“I don’t mind when someone criticizes me….of course I like to know if I’m doing right, but I’d rather know what I can improve, and how.”(IT-P18)*

Another facilitating factor was the heterogeneity in group composition. Differences in age and specialization allowed for different approaches to health problems and different models of reasoning. Because feedback providers were encouraged to clarify improvement feedback with clear examples of desired behavior, they discovered new reasoning perspectives and performance alternatives.

“*For example, we have a specialist in haptonomy in our team, and he brings in new perspectives on health problems….I profit from his views in my daily practice. For example, I try to keep the global overview instead of focusing on a single vertebra. As a manual therapist I tend to focus on the details and lose the whole picture.” (IT-P14)*

### Receiving external coach feedback

In contrast to peer feedback, participants attributed the value of coach feedback to its objectivity, conciseness, and perceptiveness, rather than to its involvement with individual peers.

*“Well, the coach had an objective approach. The feedback was very practical and well summarized. Nothing more, nothing less and because the coach was new, feedback was perceived to be more objective. I also noticed that the coach was able to discover strengths in all participants.” (IT-P2)*

However, from the PT-role perspective, the presence of the coach raised performance stress in some cases.

*“We also needed to get used to her [coach]. At least, that applied to me. You need to feel a kind of safety with each other to show openly what you think and what you do. We share this safety in our group, and that allows us not to mince words. But with a strange person here, the threshold is higher, at least in my opinion”.(IT-P1)*

Facilitating behaviors from the coach included posing critical questions rather than giving straightforward answers, fostering a safe learning environment, monitoring the structure and the time-schedule of the PA process, facilitating peer feedback delivery, and strengthening group learning. Participants rejected too much interference of the coach and judgmental coach feedback.

### Receiving simulated patient feedback

Participants varied in their appreciation of simulated patient feedback, referring to the limitations of role-play. Despite its limitations, participants valued the different perspective of patient feedback.

*“While performing the assignment, I noticed that I was not always providing clear information…I previously never thought about that …I have learned now that I need to communicate more carefully, for instance when giving bad news.” (Q-P12).*

## Performing the assessor role

### Observing a peer’s performance

Participants reported that the role of assessor allowed them to mirror and model the observed performance to their own intended performance.

*“I found observing a peer’s performance very instructive because you often imagine how you would handle the situation. When you see how your colleague deals with a problem, you critically reflect on your own choices.” (Q-P19)*

Appraising the performance of a peer was not a common practice. Participants would rather discuss than assess the observed behaviors. Giving instructive feedback (according to the feedback guidelines) was perceived as difficult. It required clear reasoning strategies, arguing for quality standards of performance, and the courage to be critical.

*“Your own feedback should be carefully considered. You must clearly explain why you do or don’t agree with the feedback of your colleagues.” (Q-P20)*

## Discussion

This study aimed to explore the critical features of a PA program that was shown to be effective in a previously published randomized controlled trial. The results clearly show that participants committed the most to learning tasks related to performance in the therapist role: performing the task, receiving peer feedback, external coach feedback, and simulated patient feedback. Participants varied widely in the perceived learning value of subtasks related to performing the PT role, but agreed on the superior value of receiving peer feedback. In the next section, we will elaborate on these results.

These results point to the importance of exposing observable behavior (PA) rather than expressing intended behavior (Case Discussion). Although exposure was associated with feelings of discomfort and performance stress, its impact on awareness of professional development was not questioned. This raises the question of how feelings of discomfort and stress can affect learning and change in professional practice.

In the PT role, participants needed to make the transfer from implicit reasoning to explicit reasoning and from intentional behavior to observable behavior to allow for assessment and feedback. Bandura’s social cognitive theory emphasizes that exposure is conditional to the development of mastery experiences, and mastery experiences are the most important source of information for the development of self-efficacy beliefs. In turn, self-efficacy beliefs contribute significantly to performance improvement and motivation to change [33]. This notion is supported by the theory of planned behavior [34]. Bandura also points to the importance of the peer group in strengthening self-confidence through “vicarious” experiences provided by social models. The impact of modeling on perceived self-efficacy is strongly influenced by perceived similarity to the models (peers) and is considered to be more powerful than performance feedback [35]. Increased self-confidence might have helped participants to approach difficult tasks as challenges to be mastered rather than as threats to be avoided.

The foregoing explains how PA participants succeeded in raising self-efficacy beliefs despite feelings of performance stress, but does not explain why they showed superior test results on clinical vignettes in the trial (Table 1). High arousal levels are generally considered to have a negative impact on the quality of performance according to the Yerkes-Dodson law [36], and PA participants’ experiences supported that, as they contended that they had underperformed in the PA context. However, they must have processed the information in a way that enhanced retrieval and transfer of knowledge to the context of clinical vignettes. Studies addressing the influence of emotion on cognitive processing provide an explanation for this apparent contradiction. McConnel & Eva [37] conducted a literature review on the impact of emotion on the transfer of clinical knowledge and skills. They conceptualized emotion by two dimensions: *valence* and *arousal*. Valence refers to the emotional state (e.g. positive or negative). Arousal refers to the level of activation. One of the findings was that emotional experiences are more likely to be mulled over than non-emotional experiences. This unintentional retrieval of emotional events might have strengthened memory traces of PA participants and facilitated the transfer to new clinical problems. Another view is presented by regulatory focus theory [38], which contends that receptiveness to feedback depends on “emotional arousal” rather than “emotional valence”. Summarizing these considerations, the critical feature of PA might be attributed to the emotional involvement (either negative or positive) with performing the PT role. As feelings of failure do not contribute to the development of self-efficacy beliefs [33], successful PA implementation should allow for coping with performance stress within or between the sessions. Training in the PT role and a safe learning environment might be crucial to enable the coping process.

Performance in the assessor role was perceived as a less powerful learning experience. However, it should be noted that the assessor role and the PT role cannot be considered as independent. Observing peer performance allowed observers to model the observed behavior, which might have contributed to reducing performance stress and triggering performance improvement. On a more unconscious level, participants might have profited from the activity of the mirror neuron system [39] that is capable of shaping the observed behavior to a virtual image of their intended behavior. In appraising their peers’ performance, assessors needed to reason aloud, compare personal views with group views, and discuss performance standards. This may have provided peer assessors with the missing data for informed self-assessment [20].

Regarding the role of the external coach in providing feedback, participants ranked peer feedback higher than coach feedback although coach feedback was valued because of its objectivity, its conciseness, and its receptiveness. A comparable study on peer assessment in undergraduate PT education, in which students were asked to rank similar learning tasks, showed that students preferred teacher feedback to peer feedback [17]. Professionals did not question the quality of peer feedback compared to coach feedback, but emphasized the importance of peers being involved in their professional development process. This finding is supported by situated learning theory [40, 41], which contends that the transfer of knowledge is hampered by the gap between the learning context and application context. Delivering the implementation program within communities of practice allows for co-constructing and tailoring knowledge to the personal learning needs [41]. In this respect, the coach remained an outsider.

Although the PA program was successful regarding its aim, the adoption of the program for knowledge transfer purposes should be carefully considered.

Firstly, some participants argued that the role-play format did not adequately reflect their authentic professional behaviors. This view is understandable, but compared to passive guideline dissemination, role-play aims to facilitate the transfer of scientific evidence to clinical practice, which it did, according to participant reports. As regards the use of peer role-play (low fidelity simulation) compared to standardized patients (high fidelity simulation), research in undergraduate education shows that both tools provide a psychological safe area of practice, where mistakes are not critical [42]. Studies on student perceptions show that standardized patients are perceived as more effective than peers [43, 44]. However, research evidence on learning outcomes remains inconclusive [44, 45]. Compared to direct observation (work-place based assessment), the role-play format allows for standardizing the content of interest, creating an adequate case mix, and describing the key-features of health problems relevant to the guidelines, [46]. Considering constraints in time and costs, peer role-play is the most feasible method. This conclusion is supported by a systematic review undertaken by Overheem et al. [47], who evaluated the feasibility and effectiveness of six methods to assess physician performance.

Secondly, some participants perceived the tight time schedule as stressing and preventing in-depth elaboration of the clinical problems. The PA program was designed to enhance the transfer from the learning context to the application context, as the transfer from one problem to another problem [48]. Yet, in an attempt to solve all the presented problems within time limits, the approach to learning might have been too superficial.

Thirdly, performance in the PT role was perceived as challenging and sometimes even threatening. When conditions of psychological safety are not met, the effectiveness of PA might be questioned [14].

## Strengths and limitations

This study provided rich data and convincing results. Because we clearly described the program design, its underlying theoretical constructs, and the critical features of successful guideline implementation, future program designers may profit from our results.

It can be argued that a limitation of the PA approach is the role-play of peers simulating patients. Although the choice of peers instead of standardized patients was defensible as argued above, and although the results show that their feedback was valued, additional training in the patient role might have increased the fidelity of the peers’ performance.

Another limitation concerns the questionnaire and the interview guide. Questionnaire comments were reduced by the three tasks with the highest-ranking results. We compensated for this limitation by interviewing participants with contrasting ranking results. Nevertheless, because we did not focus on less instructive tasks in our interviews, we might have lost information that would have underpinned our results.

Finally, the generalizability of our results might be limited because all participants in this study were Dutch. Research shows that effective peer assessment practices are culture dependent [23, 24].

## Conclusions

The effectiveness of PA can be attributed to the structured and performance-based design of the program. Participants showed a strong cognitive and emotional commitment to performing the tasks related to the physical therapist role. That might have contributed to an increased awareness of strengths and weaknesses, and a motivation to change routine practice in the management of patients with low back pain.

Conditional to successful implementation is an environment where mistakes can easily be made, but in which the self-confidence of participants remains undamaged. Adjustment of the tight time schedule and the number of cases, providing more time to elaborate on problems and to recuperate from experiences, might improve the PA task design. However, attempts to improve the effectiveness of PA should not be limited to the modification of the PA tool. We recommend a shift in the feedback culture of PTs in primary care, from avoiding performance feedback to actively seeking feedback.

Future research should address the feasibility of PA as a tool to enhance bottom-up quality improvement and accountability to external stakeholders of PT care.

## Additional files

Additional file 1: **Online questionnaire.**(PDF 213 kb) Additional file 2: **Interview guide.** (PDF 317 kb)
