# Supplementary material for: Critical features of peer assessment of clinical performance to enhance adherence to a low back pain guideline for physical therapists: a mixed methods design
Source: BMC Med Educ. 2015 Nov 12;15:203. doi: 10.1186/s12909-015-0484-1 (PMC4643538; doi:10.1186/s12909-015-0484-1)
Supplement: Additional file 1: — Online questionnaire.(PDF 213 kb) [file 12909_2015_484_MOESM1_ESM.pdf]

Online questionnaire

1. The PA program consisted of several parts. The overview below shows the distinct learning tasks and subtasks. Please rank the eleven subtasks as presented from high to low learning value (1 = most learning value, 11 = least learning value).

Overview tasks and subtasks				
**Tasks**			**Subtasks**	**Rank**
Prepare Task	Study manual	1	Study PA procedure and guidelines	
Perform Task	Perform in PT role	2	Perform clinical task individually	
		3	Receive peer feedback	
		4	Receive external coach feedback	
		5	Receive simulated patient feedback	
		6	Receive written feedback and scores	
	Perform in assessor role	7	Observe peer performance	
		8	Provide oral feedback	
		9	Provide written feedback and scores	
	Perform in patient role	19	Simulate patient problem	
Evaluate Task		11	Design and discuss change plan	

2. Please motivate your choice for the three most instructive learning tasks.

Rank	Comment*
1	
2	
3	
	*Characters unlimited
