# Supplementary material for: Critical features of peer assessment of clinical performance to enhance adherence to a low back pain guideline for physical therapists: a mixed methods design
Source: BMC Med Educ. 2015 Nov 12;15:203. doi: 10.1186/s12909-015-0484-1 (PMC4643538; doi:10.1186/s12909-015-0484-1)
Supplement: Additional file 2: — Interview guide. (PDF 317 kb) [file 12909_2015_484_MOESM2_ESM.pdf]

Interview guide

1) **What did you expect of the Peer Assessment (PA) program?**
    - Did you have personal learning goals? If so, can you describe them?
    - To what extent this program has met your expectations? Please explain.
    - The PA program aimed to enhance clinical performance of physical therapists in primary care. What are the strengths and weaknesses of PA, and why?

2) **How did you perceive the PA program, and how did it affect your daily practice?**
    - How did you perceive the PA sessions?
    - Can you remember a particular event that impressed you? If so, please describe.
    - When you look back on the PA process, did it affect your professional practice? If so, can you explain what has changed?
    - Do you think the PA process affected the professional practice of your colleagues? If so, can you explain what has changed?

3) **Which elements of the PA program to have the strongest learning value?**
The PA program consisted of several parts. The overview below shows the learning tasks and subtasks. In the questionnaire you were asked to rank eleven subtasks as presented in the overview, from high to low learning value. You indicated that you perceived task *X* to have the strongest learning value. Can you explain why? Can you proceed to do the same for task *Y* and *Z*?

**Overview of learning tasks and subtasks**			
**Tasks**			**Subtasks**
Prepare Task	Study manual	1	Study PA procedure and guidelines
Perform Task	Perform in PT role	2	Perform clinical task individually
		3	Receive peer feedback
		4	Receive external coach feedback
		5	Receive simulated patient feedback
		6	Receive written feedback and scores
	Perform in assessor role	7	Observe peer performance
		8	Provide oral feedback
		9	Provide written feedback and scores
	Perform in patient role	19	Simulate patient problem
Evaluate Task		11	Design and discuss change plan
